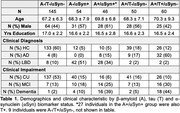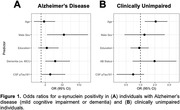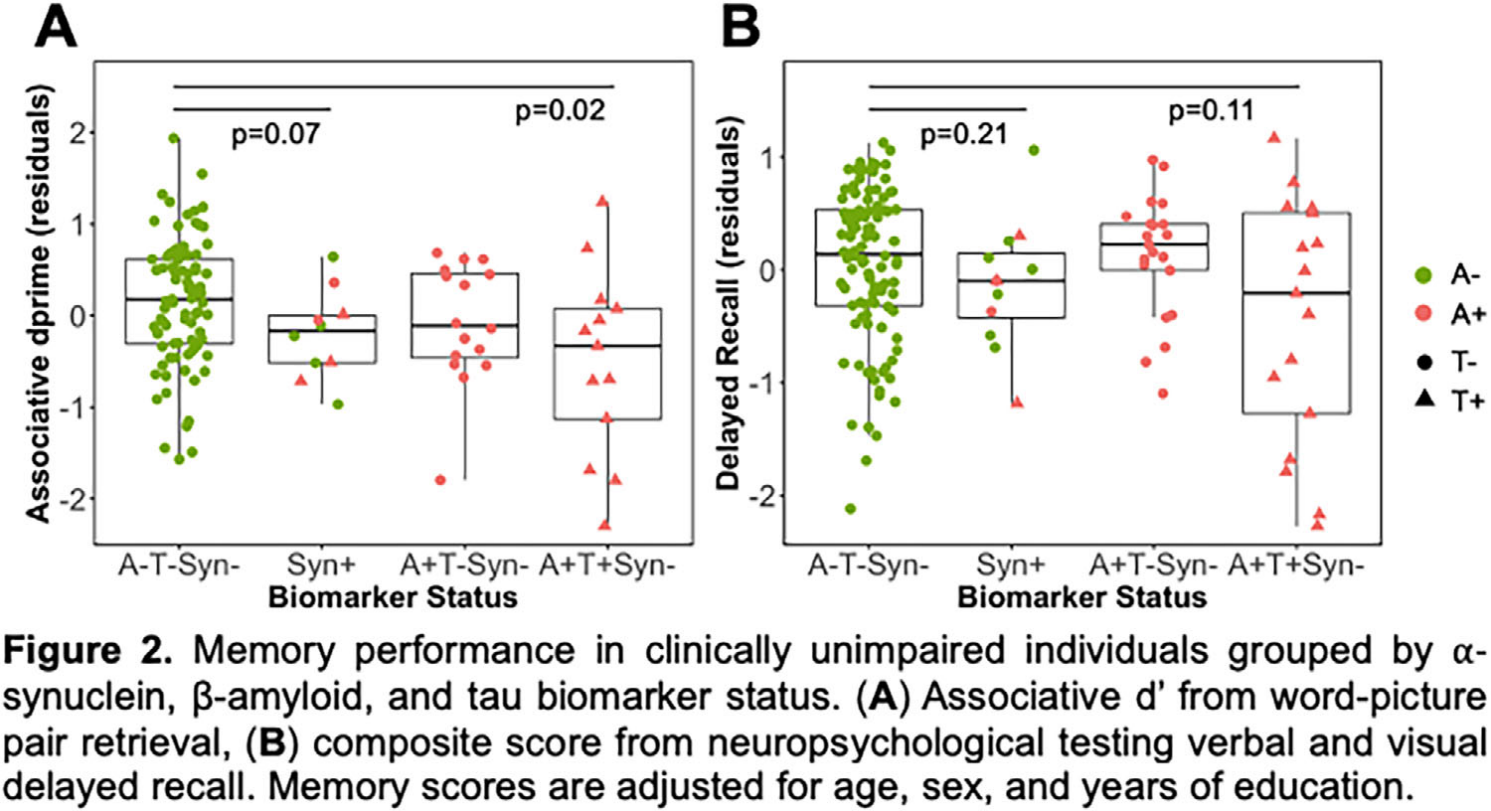# Effects of α‐synuclein pathology in Alzheimer’s disease and normal aging

**DOI:** 10.1002/alz.095792

**Published:** 2025-01-09

**Authors:** Joseph R. Winer, Melanie J. Plastini, America Romero, Edward N. Wilson, Christina B. Young, Alexandra N. Trelle, Victor W. Henderson, Anthony D. Wagner, Kathleen L. Poston, Elizabeth Mormino

**Affiliations:** ^1^ Stanford University School of Medicine, Stanford, CA USA

## Abstract

**Background:**

α‐Synuclein is the hallmark pathology of Parkinson’s disease and dementia with Lewy bodies, described together as Lewy body disease (LBD). α‐Synuclein is also commonly observed in the context of Alzheimer’s disease (AD). Here we investigate the frequency of α‐synuclein positivity in an AD research cohort and clinically unimpaired individuals (CU), as well as associations with demographics and AD biomarkers.

**Method:**

We assessed α‐synuclein status (αSyn±) in 69 clinically diagnosed AD (38 mild cognitive impairment and 31 dementia) and 277 CU using a cerebrospinal fluid α‐synuclein seeding aggregation assay. 91 LBD spectrum participants were included for comparison. Aβ status and tau levels were measured with cerebrospinal fluid Aβ42/40 and pTau181 using the Lumipulse G assay. Memory performance was examined in CU individuals enrolled in the Stanford Aging and Memory Study (SAMS, N = 164/277).

**Result:**

As expected, αSyn+ was highest in LBD (81%). 16% of AD and 9% of CU were αSyn+. Among αSyn+ CU, 45% were Aβ+ compared to 31% in αSyn‐ CU. In a logistic regression that included only participants with AD, α‐synuclein positivity was associated with male sex, clinical impairment (dementia versus mild cognitive impairment), and higher pTau181 (**Figure 1A**). In CU, α‐synuclein positivity was associated with older age (**Figure 1B**). Among CU from SAMS, the αSyn+ group showed cross‐sectional memory reductions that were similar to the A+/T+ CU group (**Figure 2**).

**Conclusion:**

α‐Synuclein status may be an important independent predictor of clinical impairment among individuals on the AD spectrum, as well as subtle memory changes in in aging.